# Establishment of Prostate Tumor Growth and Metastasis Is Supported by Bone Marrow Cells and Is Mediated by PIP5K1α Lipid Kinase

**DOI:** 10.3390/cancers12092719

**Published:** 2020-09-22

**Authors:** Richard Karlsson, Per Larsson, Regina Miftakhova, Azharuddin Sajid Syed Khaja, Martuza Sarwar, Julius Semenas, Sa Chen, Andreas Hedblom, Tianyan Wang, Kristina Ekström-Holka, Athanasios Simoulis, Anjani Kumar, Niels Ødum, Thomas Grundström, Jenny L. Persson

**Affiliations:** 1 Division of Experimental Cancer Research, Department of Translational Medicine, Lund University, Clinical Research Centre, 205 02 Malmö, Sweden; richard.karlsson@umu.se (R.K.); martuza.sarwar@umu.se (M.S.); julius.semenas@umu.se (J.S.); andreas.hedblom@umu.se (A.H.); 2 Department of Molecular Biology, Umeå University, 901 87 Umeå, Sweden; per.larsson@umu.se (P.L.); regina.miftakhova@umu.se (R.M.); sk.azharuddin@umu.se (A.S.S.K.); sa.chen@umu.se (S.C.); tianyan.wang@umu.se (T.W.); anjani.kumar@umu.se (A.K.); thomas.grundstrom@umu.se (T.G.); 3 Department of Immunology and Microbiology, University of Copenhagen, DK2200 Copenhagen, Denmark; ndum@sund.ku.dk; 4 Department of Genetics, Kazan Federal University, Kazan 420010, Russia; 5 Center for Molecular Pathology, Lund University, 205 02 Malmö, Sweden; kristina.ekstrom-holka@med.lu.se; 6 Department of Clinical Pathology and Cytology, Skåne University Hospital, 205 02 Malmö, Sweden; athanasios.simoulis@med.lu.se; 7 Department of Biomedical Sciences, Malmö University, 205 02 Malmö, Sweden

**Keywords:** prostate cancer metastasis, bone marrow cells, PIP5K1α, therapeutic interventions

## Abstract

**Simple Summary:**

Metastatic castration-resistant PCa (mCRPC) is a clinically highly lethal disease; the mechanisms underlying the lethal disease remain poorly understood. Furthermore, no effective treatment for cancer metastasis exists. In this study, we have demonstrated that prostate cancer cells required bone marrow-derived cells for their growth, survival and metastasis to the host bone marrow. Our findings have provided new evidence suggesting that cancer cell-specific signals may mediate interactions between prostate cancer cells and bone marrow cells during progression of mCRPC. Therapeutic interventions using a selective inhibitor of lipid kinase PIP5K1α may not only inhibit the growth of primary tumors but may also target the lethal mCRPC within tumor-microenvironment.

**Abstract:**

Cancer cells facilitate growth and metastasis by using multiple signals from the cancer-associated microenvironment. However, it remains poorly understood whether prostate cancer (PCa) cells may recruit and utilize bone marrow cells for their growth and survival. Furthermore, the regulatory mechanisms underlying interactions between PCa cells and bone marrow cells are obscure. In this study, we isolated bone marrow cells that mainly constituted populations that were positive for CD11b and Gr1 antigens from xenograft PC-3 tumor tissues from athymic nu/nu mice. We found that the tumor-infiltrated cells alone were unable to form tumor spheroids, even with increased amounts and time. By contrast, the tumor-infiltrated cells together with PCa cells formed large numbers of tumor spheroids compared with PCa cells alone. We further utilized xenograft athymic nu/nu mice bearing bone metastatic lesions. We demonstrated that PCa cells were unable to survive and give rise to colony-forming units (CFUs) in media that were used for hematopoietic cell colony-formation unit (CFU) assays. By contrast, PC-3M cells survived when bone marrow cells were present and gave rise to CFUs. Our results showed that PCa cells required bone marrow cells to support their growth and survival and establish bone metastasis in the host environment. We showed that PCa cells that were treated with either siRNA for PIP5K1α or its specific inhibitor, ISA-2011B, were unable to survive and produce tumor spheroids, together with bone marrow cells. Given that the elevated expression of PIP5K1α was specific for PCa cells and was associated with the induced expression of VEGF receptor 2 in PCa cells, our findings suggest that cancer cells may utilize PIP5K1α-mediated receptor signaling to recruit growth factors and ligands from the bone marrow-derived cells. Taken together, our study suggests a new mechanism that enables PCa cells to gain proliferative and invasive advantages within their associated host microenvironment. Therapeutic interventions using PIP5K1α inhibitors may not only inhibit tumor invasion and metastasis but also enhance the host immune system.

## 1. Introduction

Bone marrow-derived cells, in particular, immune-suppressive myeloid cells, have been shown to accumulate at primary tumor sites following tumor progression, leading to an increased risk for cancer metastasis to distant organs [1,2]. A substantial number of clinical data have shown that the compositions and fractions of infiltrating immune cells within primary tumors correlate with clinical outcome in various types of cancer such as breast cancer and colon cancer [1,3,4]. The presence of inflammatory infiltrators in the tumor microenvironment is now recognized as a hallmark of cancer and cancer metastasis [5]. Prostate cancer cells may utilize infiltrating bone marrow-derived cells and blood vessels to disseminate and transport to distant organs [6,7,8]. Furthermore, metastatic cancer cells are capable of inducing the expression of extracellular matrix molecules such as CXCLs and MMP9 in the tumor microenvironment for promoting distant metastasis [9,10].

It has been reported that Gr-1+CD11b+ cells, also termed myeloid immune suppressor cells (MISCs) or myeloid-derived suppressor cells (MDSCs), are significantly overproduced in the bone marrow and spleens of tumor-bearing mice [11,12] and in the peripheral blood of cancer patients [13]. CD11b+ cells were able to promote breast tumor growth and metastasis in mouse models [14]. These findings may shed light for improving current immunotherapy to target the tumor microenvironment. However, the mechanisms that mediate interactions between immune cells and cancer cells during cancer progression remain poorly understood.

PIP5K1α belongs to the PIP5K family of lipid kinases [15]. It is predominantly located close to the inner leaflet of the plasma membrane, and its enzymatic function is to convert phosphatidylinositol 4-phosphate (PIP) to phosphatidylinositol 4,5-bisphosphate (PIP_2_). We previously reported the discovery of ISA-2011B, a PIP5K1α inhibitor that selectively inhibited prostate cancer growth and invasion by targeting PI3K/AKT pathways [16,17,18]. PIP5K1α acts upstream of AKT [16,19], one of the most frequently altered proteins in human cancers. A loss of PTEN function, leading to aberrant AKT activation, is observed in 70% of all metastatic prostate cancers (PCas) [20,21].

Here, we show that bone marrow cells support the growth of primary tumors and promote the bone metastasis of PCa tumors. Tumor cell-specific signals may play an important role in recruiting bone marrow-derived cells and creating a tumor microenvironment enabling metastatic events to occur. We further showed that the inhibition of PIP5K1α reduced the ability of PCa cells to grow in the presence of myeloid cells, suggesting that tumor cells and the tumor microenvironment may be targeted by using selective kinase inhibitors. Therapeutic interventions using PIP5K1α inhibitors may not only inhibit tumor invasion and metastasis but also enhance the host immune system.

## 2. Results

### 2.1. The Functional Consequences of the Interaction between PCa Cells and a Bone-Marrow Cells

**Figure 1:**
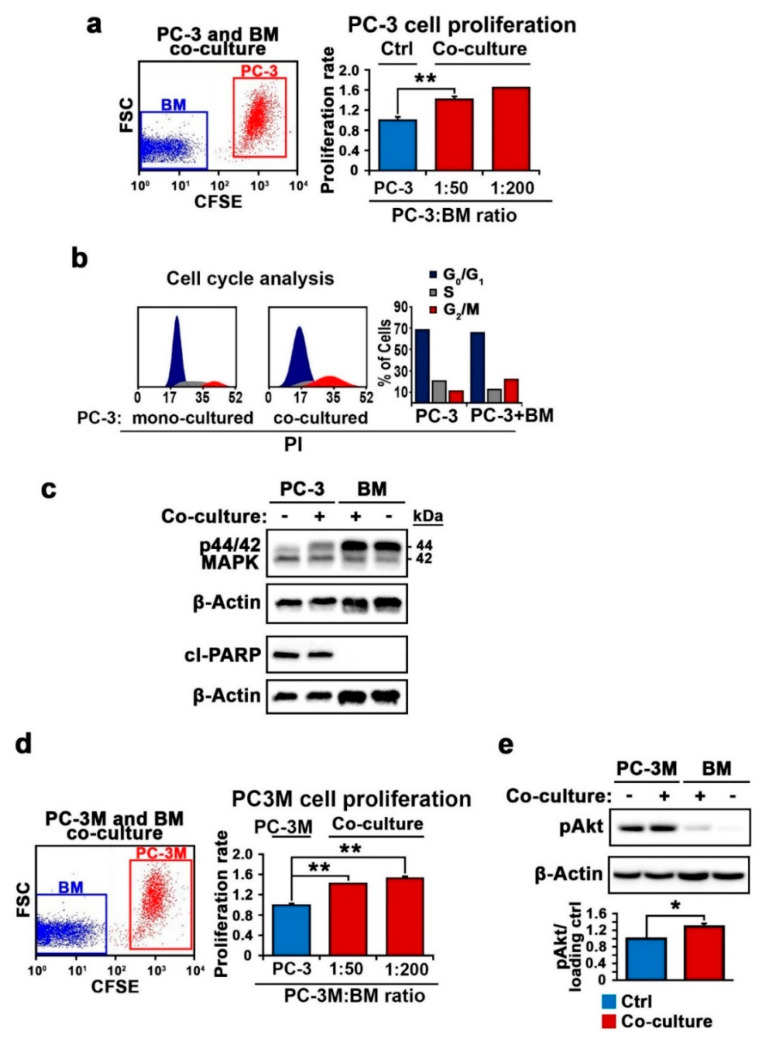
The effect of bone marrow cells on the proliferation of prostate cancer (PCa) cells. (**a**) The effect of the bone marrow cells on the proliferation of the PC-3 cells was determined using flow cytometry analysis. Representative fluorescence-activated cell sorting (FACS) plots show the separation of PC-3 and bone marrow cell (BM) populations in the left panel. The ratios between the PC-3 cells and mouse primary BM are indicated. For a ratio of 1:50 (PC-3/BM), the mean proliferation rate of the mono-cultured control PC-3 cells is set as 1, The *p*-value indicates the difference in proliferation between the PC-3 cells from the mono-culture and those from the co-culture. ** *p* = 0.002. (**b**) Cell cycle analysis of PC-3 cells from the mono-culture or co-culture was performed using flow cytometry analysis. Representative FACS histogram is shown in the left panel. The bar chart shows the distributions of the proportions of PC-3 cells at each cell cycle phase. (**c**) Immunoblot analysis of PC-3 cells vs. BM from the mono-cultured controls or co-cultured counterparts was performed using antibodies against MPAPK phor44/42, cleaved PARP and β-actin as loading controls. PC-3 cells vs. BM were collected from the same co-culture after co-culturing for 48 h at a ratio of 1:150 (PC-3/BM). (**d**) The effect of the bone marrow cells on the proliferation of PC-3M cells was determined using flow cytometry analysis. Representative FACS plots show the separation of PC-3M and BM cell populations in the left panel. Bar chart shows the PC-3M cells after 48 h of mono-culture or co-culture with BM at ratios of 1:50 and 1:200 (PC-3M/BM). For both ratios, *p* < 0.001 as indicated with “**”. Data are presented as averages of three experiments. (**e**) Expression of pAKT was determined by immunoblot analysis. Mean expression of pAKT in mono-cultured PC-3M cells was set as 1, and in co-cultured PC-3M, it was 1.29; difference = 0.29; 95% CI = 1.16 to 1.43; *p* = 0.049. * *p* < 0.05. ** *p* < 0.01. Data are presented as averages of three experiments.

We have previously reported that PCa cells preferentially home to the bone marrow microenvironment to establish metastatic growth once entering into systemic circulation [22]. To study the role of bone marrow cells in mediating the growth and progression of PCa and its underlying mechanisms, we established an in vitro co-culture system allowing the physical interactions of PCa cell lines with primary bone marrow cells from immunocompetent mice to mimic the in vivo conditions. PC-3 cells that lack a functional androgen receptor (AR), representing castration-resistant PCa cells, were employed. To distinguish PC-3 cells from the bone marrow cells and to determine the proliferation rate of the PC-3 cells, we labelled the PC-3 cells with a membrane tracer and carboxyfluorescein diacetate succinimidyl ester (CFSE) before subjecting the PC-3 cells or bone marrow cells to mono-culture or co-cultures at ratios of 1:50 and 1:200 (PC-3/bone marrow cells) for 48 h. The numbers of viable cells of two distinct populations—PC-3 cells and the bone marrow cells—within the co-culture were determined using flow cytometry analysis (Figure 1a). We observed that the proliferation rate of the PC-3 cells from the co-culture was significantly higher than that of the mono-cultured PC-3 cells after 48 h, as determined using flow cytometry analysis (for 1:50, *p* = 0.003) (Figure 1a), suggesting that the bone marrow cells are able to promote growth of PC-3 cells. Next, we subjected the PC-3 cells from the co-culture or mono-culture to the cell cycle analysis using flow cytometry. We observed that there was a significant increase in the proportion of cells at the onset of the G2–M phases in the PC-3 cells from the co-culture, compared with that of the cells from the mono-culture (Figure 1b). These data suggest that PC-3 cells undergo active proliferation in the presence of the bone marrow cells. Consistent with this, the expression of phosphorylated MAP-kinase, a key upstream regulator of the proliferation pathway, was increased in the PC-3 cells from the co-culture compared with that of the mono-cultured controls, while the expression of activated PARP, an apoptotic marker, remained similar in the PC-3 cells from the both conditions (Figure 1c and Figure S1), indicating that the rate of apoptosis was similar between the cells cultured under the two conditions.

We have previously reported that PC-3M cells consist of a high proportion of stem-like cancer cells and thus have high metastatic potential to invade the bone/bone marrow after being intravenously implanted into irradiated nude mice [22]. To investigate whether the bone marrow cells may support the growth of PC-3M cells with high metastatic potential, we subjected PC-3M cells to mono-culture or co-culture with the bone marrow cells using the same conditions as those used for PC-3 cells. Similar to what was observed for PC-3 cells, the proliferation rate of PC-3M was significantly increased after co-culture with the bone marrow cells, as compared to that of mono-cultured controls (for both PC-3M/BM cell ratios, 1:50 and 1:200, *p* < 0.001, Figure 1d). Furthermore, the expression of Ser437-pAKT, a key regulator of the PI3K/AKT pathway that is responsible for the survival and metastasis of PCa cells, was significantly increased for the PC-3M cells in the co-culture compared with that for the mono-cultured cells (*p* = 0.049, Figure 1e and Figure S1).

**Figure 2:**
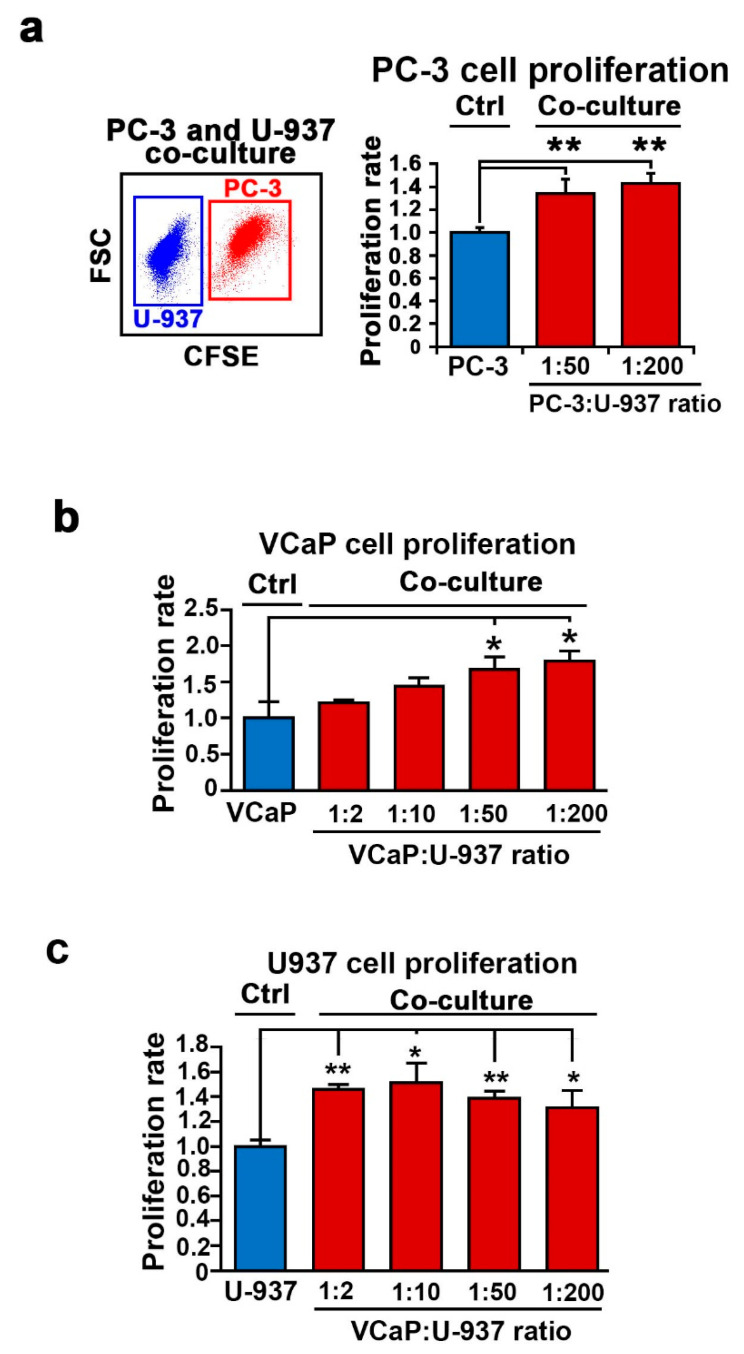
The effect of U-937 cells on the proliferation of PCa cells. (**a**) The effect of U-937 cells on PC-3 cells in co-culture was determined using flow cytometry analysis. Representative image of FACS plots showing distinct PC-3 and U-937 populations in the left panel. The bar chart shows the difference between the proliferation of PC-3 cells from the mono-cultured control and co-cultures. For the ratio 1:50 (PC-3/U-937), *p* = 0.005. For the ratio 1:200, *p <* 0.001. Data means are of duplicates from two independent experiments, shown with upper 95% confidence intervals. ** *p* < 0.01 is indicated. (**b**) The effect of U-937 cells on VCaP cells at ratios of 1:2, 1:10, 1:50 and 1:200 is shown. Difference in proliferation of VCaP cells between mono-culture and co-culture is indicated. For the ratio of 1:50 (VCaP/U-937 cells), *p* = 0.03; for the ratio of 1:200, *p* = 0.014. (**c**) The effect of VCaP cells on the proliferation of U-937 cells at ratios of 1:2, 1:10, 1:50 and 1:200 is shown. The U-937 cells were collected from the same co-culture as the VCaP cells as mentioned above in (**b**) *p* value indicates the difference in proliferation between mono-cultured U-937 cells and co-cultured U-937 cells at different ratios (VCaP/U-937 cells). For the ratio 1:2, *p* < 0.001; 1:10, *p* = 0.01; 1:50, *p* = 0.002; 1:200, *p* = 0.038. *p* < 0.05 is indicated as “*”, *p* < 0.01 is indicated as “**”.

We have previously reported that U-937 cells of myeloid cell origin are an ideal model for studying the properties of myeloid cells that express several specific cell-surface markers such as CD11b and CD11c [23]. We subjected PC-3 cells and U-937 cells to the mono-culture and co-culture assays using the same conditions as described for PC-3 and bone marrow cells. Similar to what was observed for co-culture with the bone marrow cells, the presence of U-937 cells in co-culture with PC-3 cells led to a significant increase in PC-3 cell proliferation compared with the mono-cultured controls (for the PC-3/U-937 cell ratio of 1:50, *p* < 0.001; for the ratio of 1:200, *p* = 0.003) (Figure 2a). Next, VCaP, another representative castration-resistant PCa cell line that expresses AR was subjected to co-culture assays using ratios ranging from 1:2 up to 1:200 (Figure 2b). Similar to what was observed for PC-3 cells, the presence of U-937 cells in co-culture resulted in a remarkable increase in the proliferation of VCaP cells (VCaP/U-937) (for ratio = 1:50, *p* = 0.03; for ratio = 1:200, *p* = 0.014) (Figure 2b). To examine whether PCa and U-937 cells may mutually affect each other’s growth in co-culture, we determined the proliferation rate of U-937 cells from the co-culture using flow cytometry analysis. Interestingly, the presence of VCaP cells also led to a significant increase in the growth of U-937 cells at VcaP/U-937 ratios ranging from 1:2 up to 1:200 (Figure 2c; for ratio 1:2, *p* < 0.001; for ratio 1:10, *p* = 0.01; for ratio 1:50, *p* = 0.02; for ratio 1:200, *p* = 0.038). This suggests that PCa cells also affect the growth of U-937 cells to increase their proliferative ability.

### 2.2. Tumor-Associated Myeloid Cells Promote Tumor Growth and Expansion in Xenograft Mouse Model

**Figure 3:**
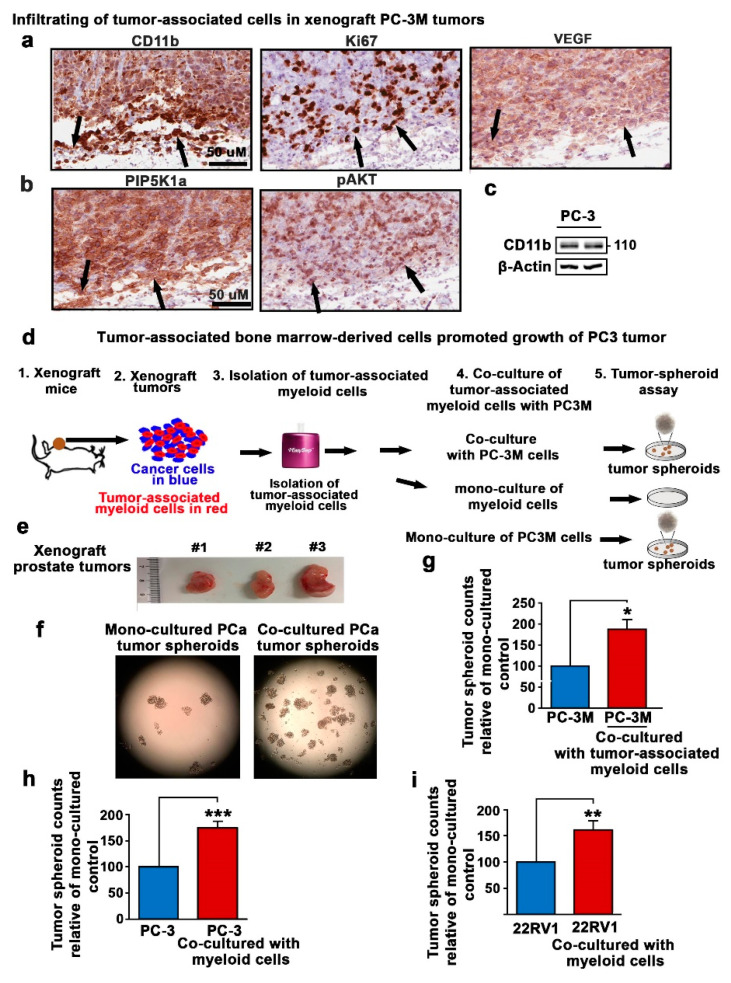
The effect of tumor-associated bone marrow-derived cells on the growth of tumor spheroids. (**a**,**b**) Immunohistochemical analysis of PC-3 xenograft tumors from mice using antibodies against CD11b, Ki67, pAKT, PIP5K1α and VEGF. Representative microphotographs of the tumor tissues stained with the abovementioned antibodies are shown. (**c**) Immunoblot analysis of CD11b expression in PC-3 cells. Antibody against human CD11b was used. β-actin was used for loading controls. (**d**) The schematic illustrations show the procedures for the preparation of tumor-infiltrated myeloid cells from xenograft PC-3 tumors from mice. Tumors were dissected and made into single-cell suspensions. The Lin+ cells were isolated by using an EasySep kit for the isolation of mouse hematopoietic cells from bone marrow. The Lin+ cells and PC-3M cells were subjected to mono-culture or co-culture for tumor-spheroid formation assays. The spheroids were counted and quantified after 14 days. (**e**) Representative image of the tumors that were used for the isolation of Lin+ cells is shown. (**f**) Representative images of the tumor spheroids derived from mono-cultured PC-3M or PC-3M and Lin+ cells are shown. (**g**) A bar chart indicating the relative counts of tumor spheroids derived from mono-cultured PC-3M or PC-3M and Lin+ cells co-cultured together is shown (*p* = 0.009). (**h**) A bar chart indicating the relative counts of tumor spheroids derived from mono-cultured PC-3 cells or PC-3 and Lin+ cells co-cultured together is shown (*p* < 0.001 is indicated as “***”). (**i**) A bar chart indicating the relative counts of tumor spheroids derived from mono-cultured 22Rv1 cells or 22Rv1 cells and Lin+ cells co-cultured together is shown (*p* = 0.01). *p* < 0.05 is indicated as “*”, *p* < 0.01 is indicated as “**”.

The possibility that bone marrow-derived cells may contribute to tumor invasion and metastasis led us to examine whether these cells infiltrate into primary tumors. We utilized a xenograft mouse model bearing human PC-3 tumors from subcutaneous implantation [16]. This tumor model shares many characteristic features with human castration-resistant PCa, particularly its ability to form large tumors over 500 mm^3^ after 30 days of tumor inoculation. To examine the role of the myeloid cells in mediating tumor progression, tumor tissues were collected when the mean tumor volumes reached over 500 mm^3^. We stained tumor tissues using immunohistochemical analysis with anti-CD11b antibody, the marker for myeloid cells. Interestingly, a large proportion of tumor tissues exhibited a high level of CD11b expression (Figure 3a). In addition to CD11b-positive cells, we observed that Ki-67, a proliferation marker, was highly expressed throughout the tumor tissues (Figure 3a). The high proportion of CD11b-positive cells was coincident with the high level of expression of VEGF, PIP5K1α and Ser437-pAKT, markers for tumor survival, angiogenesis and metastasis in the center region and in the invasive front of the tumor tissues (Figure 3b). Interestingly, immunoblot analysis revealed the presence of CD11b expression in PC-3 cells (Figure 3c and Figure S1). To examine whether the PC-3 xenograft tumors contained mixtures of CD-11b-positive (CD11b+) tumor cells and tumor-associated CD11b+ myeloid cells from the mouse host, we performed hematopoietic cell purification assays to isolate mouse CD11b+ and Gr1+ myeloid populations from the PC-3 xenograft tumors using a mouse-hematopoietic lineage-specific EasySep kit (Figure 3d,e). This kit allowed the purification of mouse myeloid cells that infiltrated into the human xenograft tumors as described in our previous studies [24]. We then subjected the purified CD11b+ and Gr1+ cells to a tumor-spheroid formation assay that was designed specifically for tumor-initiating cells (Figure 3d,e).

Interestingly, CD11b+ and Gr1+ cell populations isolated from the PC-3 xenograft tumors or mouse bone marrow did not give rise to tumor spheroids in semi-dry spheroid media under different conditions. By contrast, PCa cells including PC-3M, PC-3 and 22RV1 cells alone gave rise to tumor spheroids (Figure 3f–i). The presence of CD11b+ bone marrow-derived cells in PC-3 xenograft tumors led us to examine whether these infiltrated myeloid cells may mediate tumor progression when co-existing with cancer cells. Remarkably, PC-3M co-cultured together with bone marrow-derived cells from PC-3 xenograft tumors generated significantly higher numbers of tumor spheroids as compared to those of mono-cultured PC-3M cells (*p =* 0.009, Figure 3g). Similar to what was observed for PC-3M cells, PC-3 cells co-cultured together with CD11b+ and Gr1+ cell populations generated significantly higher numbers of tumor spheroids as compared to those of mono-cultured controls (*p* < 0.001, Figure 3h). To further study the biological relevance, we applied 22Rv1 cells that express androgen receptor (AR) and represent metastatic castration-resistant PCa. Similarly to PC-3M and PC-3 cells, 22Rv1 cells co-cultured with CD11b+ and Gr1+ cell populations produced higher numbers of tumor spheroids compared with the mono-cultured controls (*p* = 0.01, Figure 3i). These data suggest that the host bone marrow cells are able to enhance tumor growth.

### 2.3. Interaction between PCa Cells and Bone Marrow Cells Correlates with Increased BM Metastasis in a Xenograft Mouse Model

**Figure 4:**
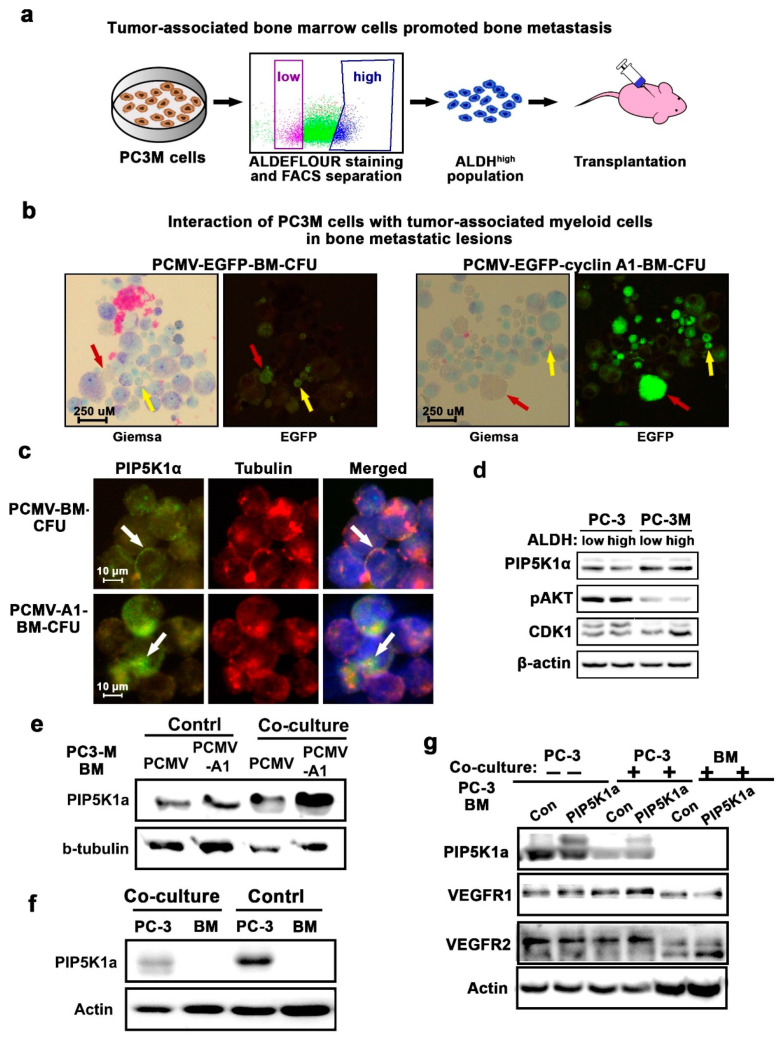
The effect of bone marrow cells on PCa bone metastasis. (**a**) A schematic chart depicts the procedure of transplantation of FACS-sorted ALDH^high^ PC-3M cells expressing pCMV-EGFP or pCMV-EGFP-cyclin A1 into nude mice through intra-cardiac injection. *n* = 7 mice per group. (**b**) A colony-forming unit (CFU) assay was performed. Cell suspensions were collected from the CFU colonies derived from the bone marrow of mice that received ALDH^high^ PC-3M cells expressing pMSCV-EGFP (pCMV-Ctrl) or (pCMV-CCNA1). Cells stained with Giemsa are shown in the left panels, and PCa cells positive for EGFP in fluorescence (green) are shown in the right panels. The cancer cells are indicated by red arrows and bone marrow cells are indicated by yellow arrows in the images. (**c**) Immunofluorescence analysis of expression and subcellular localization of PIP5K1α (FITC) in the tumor cells within the CFUs derived from bone marrow metastatic lesions expressing pCMV control vector or pCMV-cyclin A1. Antibody against tubulin highlighted the cytoskeleton of the tumor cells. (**d**) Immunoblot analysis of expression of PIP5K1α, pAKT and CDK1 in FACS-sorted ALDH^high^ and ALDH^low^ populations of PC3M cells and PC3 cells, respectively. (**e**) The effect of bone marrow cells on the expression of PIP5K1α in PC-3M cells expressing pMSCV-EGFP (pCMV-Ctrl) or (pCMV-CCNA1) that were in mono-culture or in co-culture with the bone marrow cells was determined using immunoblot analysis. Antibodies against PIP5K1α β-tubulin were used. (**f**) Expression of PIP5K1α in PC-3 cells and bone marrow cells from the mono-culture or co-culture was determined using immunoblot analysis. (**g**) The effect of PIP5K1α overexpression on VEGFR1 and VEGFR2 in PC-3 cells and bone marrow cells in the mono-culture or co-cultured together was determined using immunoblot analysis.

To further examine the critical roles of bone marrow cells in mediating bone metastasis and to gain deeper understanding of the biological consequences of the interactions between PCa cells and bone marrow cells during the establishment of PCa metastasis in the bone marrow in in vivo conditions, we utilized a PC-3M metastatic mouse model bearing bone metastasis from the intra-cardiac implantation of a subpopulation with a high level of aldehyde dehydrogenase 1 (ALDH1) isolated from PC-3M cells, which were described in our previous studies [22]. ALDH1 is a detoxifying enzyme responsible for the oxidation of intracellular aldehydes and is a marker for stem-like cancer cells. ALDH^high^ stem-like PCa cells overexpressing cyclin A1 exhibited a higher capacity to integrate into the bone marrow compared with those expressing control vector or total PC3M cells [22]. This tumor model shares many characteristics with human PCa bone metastasis; we therefore utilized this model to examine the interactions between PCa cells and bone marrow cells within metastatic lesions in vivo (Figure 4a). To this end, a 1 × 10^5^ ALDH^high^ subpopulation of PC-3M cells expressing EGFP or cyclin A1-EGFP vectors that were sorted by FACS Aria were intra-cardiacally implanted into nude mice, and bone metastasis was allowed to establish for 30 days after tumor inoculation. The metastasis was monitored and determined using an in vivo imaging device and the flow cytometry analysis of the EGFP-positive cells as described in our reported studies [22]. The bone marrows collected from the mice bearing bone metastases or tumor-free control mice were subjected to colony-forming unit (CFU) assays. As expected, no colonies were derived from PC3M cells alone in the CFU media specifically designed for hematopoietic cells, even after 14, 20 or 30 days. By contrast, bone marrows containing PC3M metastatic lesions gave rise to colonies after 14 days. Interestingly, microscopic analysis of the CFUs revealed the presence of EGFP-positive PC3M tumor cells together with the bone marrow cells (Figure 4b). There were higher numbers of EGFP-positive PC-3M cells that were in close contact with the bone marrow cells from the metastatic lesions overexpressing cyclin A1 as compared to the controls (Figure 4b). These data suggest that bone marrow myeloid cells may be critical for cancer cells to establish metastatic growth.

The constitutive expression of PIP5K1α/AKT has been shown to be associated with the growth and metastasis of PCa [16,18]. We found that PIP5K1α expression was present in the CFUs derived from the bone metastatic lesions of the mice that received PC3M cells expressing either EGFP vector or cyclin A1-EGFP (Figure 4c). We then examined the effect of cyclin A1 overexpression on PIP5K1α in PC3M from mono-culture or co-culture with bone marrow cells. As reported in our previous study, cyclin A1 expression was increased in the ALDH^high^ population of PC3M cells compared with that in the ALDH^low^ population [22]. Here, we found that the expression of CDK1, a key kinase partner of cyclin A1, was also markedly increased in the ALDH^high^ population of PC3M cells compared with that in the ALDH^low^ population (Figure 4d). The expression of PIP5K1α and pAKT remained similar in ALDH^high^ cells and their counterpart ALDH^low^ populations isolated from both PC-3M and PC-3 cells, respectively (Figure 4d and Figure S1). Interestingly, the expression of PIP5K1α was greatly increased in PC-3M cells that overexpressed cyclin A1 after being co-culturing with the bone marrow cells as compared with the controls (Figure 4e and Figure S1). We next wanted to examine whether an elevated level of PIP5K1α expression may be associated with the metastatic invasion of PCa cells into bone/bone marrow. Given that PC3M already expressed maximal levels of PIP5K1α, we used PC-3 cells as an in vitro model system and examined the effect of PIP5K1α overexpression on the downstream key regulators in PC-3 cells under mono-culture or co-culture conditions. There was no detectable PIP5K1α expression in the bone marrow cells, in contrast to that in PC-3 cells (Figure 4f and Figure S1). Interestingly, when the elevated expression of PIP5K1α was induced in PC-3 cells, the co-cultured PC-3 cells with elevated levels of PIP5K1α had slightly increased VEGFR1 expression compared with that of controls (Figure 4g and Figure S1). Furthermore, it was apparent that VEGFR2 expression increased in the bone marrow cells that were co-cultured with PC-3 cells overexpressing PIP5K1α. This observation is consistent with our previous reported studies, in which the inhibition of PIP5K1α led to significantly reduced expression of VEGFR1 and VEGFR2 [25], suggesting that PIP5K1α may be functionally linked to the VEGF-receptor signaling axis by mediating interactions between PCa cells and bone marrow cells.

### 2.4. PIP5K1α Mediates Interaction between PCa Cells and Tumor-Associated Myeloid Cells

**Figure 5:**
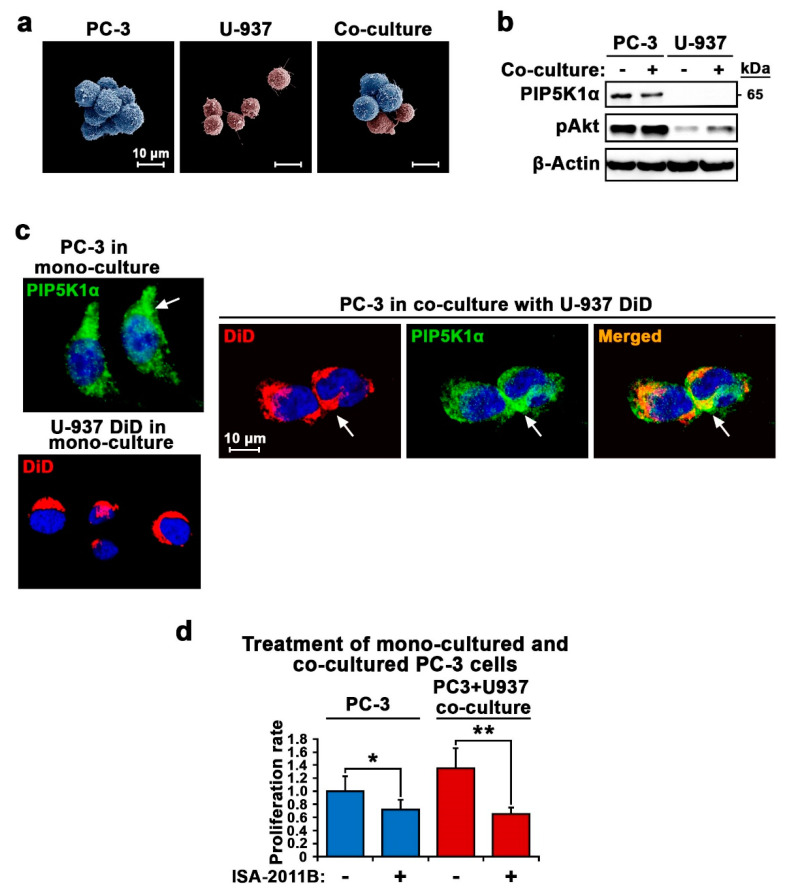
Interaction between PC-3 cells and bone marrow cells is linked to PIP5K1α-associated pathways. (**a**) Transmission electron microscopy images of U-937 cells (pink) and PC-3 cells (blue) in mono-culture or co-cultured in suspension culture conditions are shown. (**b**) Immunoblot analysis of PIP5K1α/phosphorylated AKT (pAKT) expression in PC-3 and U-937 cells in mono- (−) or in co-culture (+) is shown. (**c**) Representative immunofluorescent images showing the 1,1′-dioctadecyl-3,3,3′,3′-tetramethylindodicarbocyanine perchlorate (DiD)-membrane stained U-937 cells from mono-culture and mono-cultured PC-3 cells stained with fluorescein (FITC)-conjugated PIP5K1α antibody (left panel). U-937 cells were stained with DiD and were co-cultured with PC-3 cells. PC-3 cells were collected and were stained with FITC-conjugated PIP5K1α antibody. In the right panel, representative images show that PC-3 cells collected from the co-culture show DiD staining in red and PIP5K1α-antibody staining in green. Merged image shows the overlap of red and green in orange color. (**d**) The effect of inhibition of PIP5K1α by its kinase inhibitor ISA-2011B on PC-3 cell proliferation in mono-culture and in co-culture with U-937 cells is shown. Mono- or co-cultured PC-3 cells were treated with DMSO as control or ISA-2011B at 25 µM for 48 h. For mono-cultured control-treated vs. ISA-2011B-treated PC-3 cells, *p* = 0.031. For co-cultured control-treated vs. ISA-2011B-treated cells, *p* < 0.001. Data means are from three independent experiments. ** *p* < 0.01 and * *p* < 0.05 are indicated.

Next, we employed human bone marrow-derived monocyte U-937 cells to examine the morphology and behaviors of PCa cells in co-culture with bone marrow cells. Scanning electron microscopic analysis revealed that PC-3 and U-937 cells adhered to each other (Figure 5a). We again observed that PIP5K1α expression was absent in U-937 cells but present in PC-3 cells, and the level of PIP5K1α remained similar in PC-3 cells between two culture conditions according to immunoblot analysis (Figure 5b and Figure S1). Consistent with our findings mentioned in Figure 1, the expression of phosphorylated AKT was higher in co-cultured PC-3 cells compared with that in mono-cultured cells (Figure 5b and Figure S1). To further examine the subcellular localization of PIP5K1α and monitor the dynamics of cell–cell interaction between PCa cells and U-937 cells during the co-culture, we used 1,1′-dioctadecyl-3,3,3′,3′-tetramethylindodicarbocyanine perchlorate (DiD) in red, a membrane tracer to label U-937 cells before subjecting them to mono-culture or co-culture with unstained PC-3 cells for 24 h. Consistent with what was shown by immunoblotting, PIP5K1α expression was undetectable in U-937 cells that were stained with DiD (Figure 5c). By contrast, mono-cultured PC-3 cells showed distinct PIP5K1α staining signals in green, which were predominantly localized in the cytoplasmic/membrane compartment of the PC-3 cells (Figure 5c).

Next, we assessed whether the inhibition of PIP5K1α may block the effect of U-937 cells on PC-3 cell proliferation. To this end, we treated PC-3 cells and PC-3 cells that were co-cultured together with U-937 cells with ISA-2011B, the PIP5K1α inhibitor, or vehicle control for 48 h. Consistent with our previous reported studies [25], ISA-2011B treatment resulted in the decreased proliferation of PC-3 cells from the mono-culture (Figure 5d). Remarkably, the inhibition of PIP5K1α by ISA-2011B had a profound inhibitory effect on the proliferation of the PC-3 cells from the co-culture compared with that of the mono-cultured control (for mono-culture, *p* = 0.03; for co-culture, *p* < 0.001) (Figure 5d).

**Figure 6:**
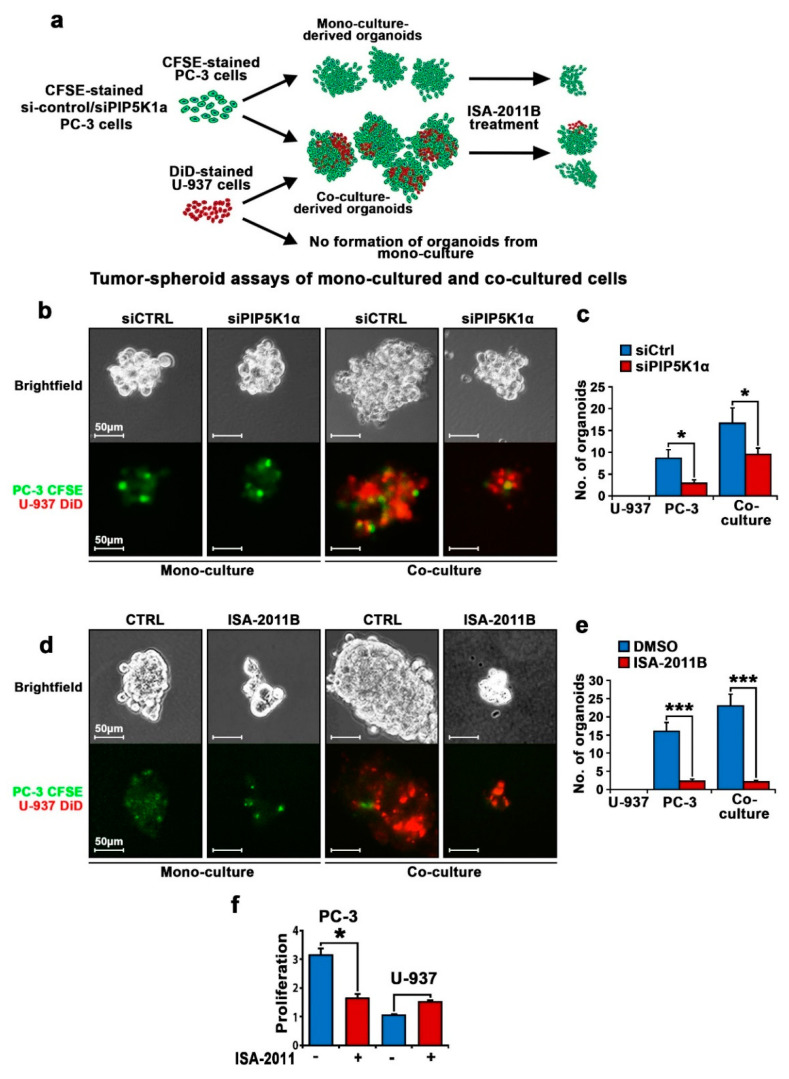
The effect of PIP5K1α inhibition on the interaction between PC-3 cells and U-937 cells. (**a**) Schematic chart depicting the experimental procedures of the establishment of PCa tumor spheroids using mono-cultured or co-cultured PC-3 cells transfected with control siRNA or siPIP5K1α RNA with U-937 cells, or mono-cultured PC-3 vs. co-cultured tumor spheroids treated with vehicle control or ISA-2011B. U-937 cells were stained with DiD and were co-cultured with PC-3 cells that were stained with carboxyfluorescein diacetate succinimidyl ester (CFSE). (**b**) Representative images of the micro-photographs of tumor spheroids taken under the confocal microscope to visualize tumor cells within tumor spheroids derived from mono-cultured si-control PC-3 cells or siPIP5K1α-PC-3 cells, or the interaction between PC-3 cells and U-937 cells in tumor spheroids derived from the co-cultured cells, are shown. (**c**) The tumor-spheroid counts from different groups are shown. Mono-culture *p* = 0.017; co-culture, *p* = 0.047. (**d**) Representative images of the micro-photographs of tumor spheroids to visualize tumor cells within tumor spheroids derived from mono-cultured or co-cultured PC-3 cells treated with vehicle control or ISA-2011B. (**e**) The tumor-spheroid counts from different groups are shown. *p* < 0.001 as indicated by “***”. (**f**) The comparison of the effect of ISA-2011B on the proliferation of PC-3 cells and U-937 cells, respectively. *p* < 0.05 as indicated by “*”.

Next, the growth-inhibitory effect of PIP5K1α inhibition on co-cultured PC-3 cells was further assessed by using tumor-spheroid assays. To distinguish PC-3 cells from U-937 cells in the flow cytometry and fluoresces-microscopic assays, U-937 cells were stained with DiD in red, while PC-3 cells were stained with a membrane tracer marker, carboxyfluorescein diacetate succinimidyl ester (CFSE), in green (Figure 6a). We inhibited PIP5K1α by transfecting siRNA for PIP5K1α or control siRNA before subjecting the mono-cultured or co-cultured cells to tumor-spheroid assays (Figure 6a). Similar to what was observed for the bone marrow cells, U-937 cells alone did not give rise to tumor spheroids (Figure 6c). By contrast, tumor spheroids were derived from the control siRNA-transfected PC-3 cells cultured together with U-937 cells (Figure 6b,c). Interestingly, DiD-positive U-937 cells were observed in the tumor spheroids (Figure 6b,c). Remarkably, the numbers and sizes of the tumor spheroids derived from siRNA-control PC-3 and U-937 cells after co-culture were significantly higher compared with those from the mono-cultured PC-3 controls (*p* = 0.029, Figure 6b). There was a significant reduction in both the sizes and amount of tumor spheroids that were derived from the co-cultured si-PIP5K1α PC-3 cells as compared with those of the siRNA controls (for mono-culture, *p* = 0.017; co-culture, *p* = 0.047; Figure 6b,c). Next, we used ISA-2011B to inhibit PIP5K1α. The PIP5K1α inhibitor ISA-2011B remarkably decreased the production of the tumor spheroids derived from mono-cultured PC-3 cells and co-cultured PC-3 and U-937 cells (for mono-culture, *p* < 0.001; for co-culture, *p* < 0.001, Figure 6d). Furthermore, the inhibitory effect of ISA-2011B was more profound on the growth of tumor spheroids derived from the co-cultured PC-3 and U-937 cells (Figure 6d,e). These data showed that a PIP5K1α inhibitor or siRNA for PIP5K1α decreased the survival and growth ability of PC-3 cells in the presence of U-937 cells. To confirm that the inhibitory effect of ISA-2011B on the growth of PCa cells was specifically through inhibiting PIP5K1α, we treated PC-3 cells and U-937 cells with ISA-2011B side by side for 48 h. As expected, ISA-2011B had no inhibitory effect on the proliferation rate of the U-937 cells, as the U-937 cells had no detectable level of PIP5K1α expression (Figure 6f). By contrast, ISA-2011B exhibited a significant inhibitory effect on the proliferation of PC-3 cells that had a high level of PIP5K1α (Figure 6f). These results are consistent with our recent reported studies showing that ISA-2011B treatment resulted in the reduced proliferation and invasiveness of PCa cells [25].

## 3. Discussion

The following remain poorly understood: (i) why the immune cells are incapable of killing tumor cells but rather form a tumor microenvironment that promotes metastasis; (ii) what the underlying mechanism that allows tumors to recruit and produce immunosuppressive factors is. In the present study, we explored the functional aspects underlying communication between PCa cells and bone marrow-derived cells during growth and metastasis.

Although the role of bone marrow cells in contributing to cancer metastasis has been previously reported, the molecular mechanisms by which tumor cells may utilize bone marrow cells remained poorly understood. Furthermore, it is largely unknown whether tumor cells may utilize the tumor-specific signals to mediate the interactions with their associated microenvironment to gain proliferative and invasive advantages. We, for the first time, show that PCa cells communicate with bone marrow cells to establish metastatic growth and survival in the host bone marrow environment. We further demonstrate that the activation of PIP5K1α/AKT signaling may play a role in the metastatic events.

In our established ex-vivo model, we utilized xenograft invasive prostate PC-3 tumors with high distant metastatic potential from athymic nu/nu mice. CD11+ and Gr1+ are known to be immunosuppressive cells. Here, we showed that both myeloid cells and PC-3 cells expressed CD11b. We observed that CD11b+ cells were most abundant in PC-3 xenograft tumors, suggesting that PC-3 xenograft tumors are a mixture of tumor cells and tumor-infiltrated myeloid cells. Furthermore, PC-3 xenograft tumors displayed high levels of PIP5K1α, phosphorylated AKT and angiogenic factor VEGF. Our findings suggest that bone marrow-derived CD11+ cells that infiltrate into tumor tissues may create an environment that favors tumor progression.

In this study, we isolated bone marrow cells that mainly constituted populations that were positive for CD11b and Gr1 antigens from xenograft PC-3 tumor tissues from athymic nu/nu mice. Since T-cell function in athymic nu/nu mice is abolished, we assumed that the immunosuppressive CD11+ and Gr1+ cells that infiltrated into the tumor tissues would play an important role in mediating tumor progression. We therefore tested our hypothesis by subjecting these cells to tumor-spheroid formation assays alone or together with PC-3M cells that were enriched in stem-like cancer cells compared with PC-3 cells. We found that the tumor-infiltrated cells alone were unable to form tumor spheroids, even with increased amounts and time. By contrast, the tumor-infiltrated cells together with PC-3M cells formed large numbers of tumor spheroids compared with PC-3M cells alone, as PC-3M cells are able to give rise to tumor spheroids. We used both PC-3-derived cells and 22Rv1 cells that represent metastatic castration-resistant PCa as the model systems. These data confirmed our findings from in vivo models, further suggesting that bone marrow-derived tumor infiltrating cells may create an environment that favors tumor progression.

In this study, we utilized xenograft mice bearing bone metastatic lesions. We tested whether PC-3M cells required bone marrow cells for their growth and survival in the host bone marrow environments. Our results showed that PC-3M cells required bone marrow cells to support their growth and survival. The PC-3M cells were unable to survive and give rise to CFUs in media that were used for hematopoietic cell colony formation. However, the PC-3M cells survived and gave rise to CFUs when bone marrow cells were present.

In cancer cells, a wide range of signaling proteins and receptors residing in lipid membranes regulate the metastatic dissemination and growth of cancer. Our present study suggests that tumor-infiltrating cells are likely to be one of the major sources of the increased PIP5K1α and AKT activity observed in PCa. We have previously shown that the overexpression of PIP5K1α signaling resulted in aggressive tumor progression in several mouse models [16,18,26]. In this study, we found that PIP5K1α was highly expressed in PCa cells but was undetectable in bone marrow cells and the U-937 myeloid cell line. Furthermore, a high level of PIP5K1α was observed in bone metastatic lesions. We assumed that PIP5K1α may be required for cancer cells to establish growth and survival in the host environment. We therefore tested our hypothesis that PCa cells that lack PIP5K1α may be unable to survive in the bone marrow host environment. Our results showed that PCa cells that were treated with either siRNA for PIP5K1α or its specific inhibitor, ISA-2011B, were unable to survive and produce tumor spheroids, in the presence of U-937 bone marrow cells. We showed that ISA-2011B only had an inhibitory effect on PCa cells from mono-culture or co-culture with U-937 cells. However, ISA-2011B had no inhibitory effect on U-937 cells that lacked a detectable level of PIP5K1α. Our findings suggest that ISA-2011B has on-target effects to inhibit the growth of PCa cells that are associated their microenvironment.

Metastatic castration-resistant PCa (mCRPC) is a clinically highly lethal disease; the underlying mechanisms that drive androgen-dependent PCa to become mCRPC remain poorly understood. Furthermore, no effective treatment for the disease exists. Our findings have provided new evidence suggesting that interactions between PCa cells and the tumor microenvironment may contribute to the progression of PCa to an mCRPC state. Therapeutic interventions using a PIP5K1α inhibitor may not only inhibit the growth of primary tumors but may also target the lethal mCRPC.

## 4. Materials and Methods

### 4.1. Cell Culture and Treatments

The PC-3 (RRID:CVCL_0035), U-937 (RRID:CVCL_0007) and VCaP (RRID:CVCL_2235) cell lines were purchased from the American Type Culture Collection (Manassas, VA, USA). An androgen-insensitive cell line, PC-3M [27], was kindly provided by Dr. J Fidler (Department of Urology, MD Andersson Cancer Center, Houston, TX, USA). The cells were maintained in RPMI-1640 medium or Ham’s F-12 medium supplemented with 10% fetal bovine serum (FBS), 1% penicillin–streptomycin–neomycin (PSN) and 2 mM l-Glutamine. All the human cell lines were purchased within the last three years or have been authenticated using short-tandem repeat (STR) profiling within the last three years. All experiments were performed with mycoplasma-free cells. PC-3, U-937 and BM cells in mono- or co-culture were treated with a PIP5K1 alpha inhibitor—ISA-2011B, a diketopiperazine-fused C-1 indol-3-yl-substituted 1,2,3,4-tetrahydroisoquinoline derivative—at a final concentration of 25 or 50 µM in 0.125% or 1% DMSO for 48 h. Cell counts were assessed by FACS analysis on a CytoFLEX analyzer (Beckman Coulter, FL, USA).

### 4.2. Co-Culture of PCa Cells with U-937 Cells or Bone Marrow Cells

PC-3 cells, PC-3M cells, VCaP cells and U-937 cells were maintained in mono-culture or in co-culture at ratios (PC-3/U-937 or PC-3M/U-937) of 1:2, 1:10, 1:50 or 1:200 for 48 h. For co-culturing cancer cells and bone marrow cells, mouse primary bone marrow (BM) cells were isolated by crushing the long bones from NMRI nude mice or C57BL/6J mice aged 3 to 5 months. The cell clumps and debris were removed by filtering the cells through a 70 μm cell strainer (BD Bioscience, MA, USA). Mono- or co-culture at ratios (PCa/BM cells) were carried out for 48 h.

### 4.3. Fluorescence-Activated Cell Sorting (FACS) and Analysis of Co-Cultured Cells

For the flow cytometry analysis and FACS sorting of co-cultured cells, cells were stained with CFSE (Invitrogen, Stockholm, Sweden) or DiD (Molecular Probes Inc. Eugene, OR, USA) according to the manufacturers’ protocols prior to co-culture. The cells were sorted using a FACSAria III (BD Biosciences, Stockholm, Sweden). Flow cytometry analysis was performed using a CytoFLEX, CyAn™ ADP analyzer (Beckman Coulter, Brea, CA, USA). The samples were analyzed using the FCS Express (DeNovo Software, Pasadena, CA, USA), FlowJo (Tree Star, Inc., Ashland, OR, USA) and CytExpert software version 2.3 (Beckman Coulter, Brea, CA, USA. The sorted cells were collected and snap-frozen in liquid nitrogen for immunoblot analysis.

### 4.4. FACS-Based Cell Cycle Analysis

The cells were stained with 50 µg/mL propidium iodide and 1 µg/mL Nonidet P40 (Sigma-Aldrich, Stockholm, Sweden), 10 µg/mL RNAse A (AppliChem, Kongens Lyngby, Denmark) and 0.58 µg/mL NaCl, and the cell cycle distribution was determined using a CytoFLEX analyzer.

### 4.5. Plasmids, Transfection and siRNA Knockdowns

For transient transfection studies, pMSCV-cyclinA1-EGFP containing the full-length (1.8 kB) human cyclin A1 cDNA and pMSCV-EGFP constructs (Clonetech Inc., Mountain View, CA, USA) were used. For inducing PIP5K1α overexpression in PCa cells, the full-length cDNA of human PIP5K1α (pLPS-PIP5K1α) or control vector (pLPS-EGFP) were constructed and transiently transfected using the transfection reagent Lipofectamine^®^ 2000 (Life Technologies, Paisley, UK) or TransIT-X2^®^ (Mirus Bio, Madison, WI, USA) according to the manufacturers’ instructions. In the PIP5K1α knockdown experiments, PCa cells were treated with *PIP5K1A*-siRNA or negative-control siRNA (VWR International Inc., Radnor, PA, USA), respectively, by using the TransIT-TKO^®^ kit according to the manufacturer’s instructions (Mirus Bio).

### 4.6. Colony-Forming Unit (CFU) Assays Using Methylcellulose-Based Medium

To assess the repopulating ability and differentiation potential of the BM progenitor cells or metastatic PCa cells in the bone marrow, a methylcellulose-based colony-forming assay was performed according to the manufacturer’s description (MethoCult™ GF M3434, Stem Cell Technologies, Vancouver, BC, Canada). Colonies were collected and microphotographs were taken after 14 days.

### 4.7. Prostate Cancer Stem-Like Cell-Derived Tumor-Spheroid Formation Assay

PC3M cells, PC-3 cells and U-937, either in mono-culture or co-culture, were subjected to tumor-spheroid assays. A total of 2000 cells from mono-cultured or co-cultured PCa and U-937 cells/BM cells at 1:2 ratios (PCa/BM) were prepared in single-cell suspensions and were seeded in a modified organoid medium containing DMEM F-12, 3.151 g/L Glucose, l-Glutamine, 2 × B27, 40 ng/mL EGF and 40 ng/mL FGFb, and were cultured in 2 mL in 35 mm polyHEMA-coated culture dishes for 14 days.

### 4.8. Mouse Model of PCa Xenograft Tumors and Distant Metastases

The animal studies were approved by the Swedish Regional Ethical Animal Welfare Committee (A3-19). The animal welfare guidelines were strictly followed. Athymic NMRI nude male mice (Taconic Europe, Lille Skensved, Denmark, or Charles River Biotechnology) aged 4–6 weeks and weighing 25–27 g were used. Two sets of mouse experiments were performed. (i) in the first set, 1 × 10^5^ ALDH^high^ PC-3M cells expressing EGFP or EGFP-cyclin A1 [22] were implanted via intra-cardiac injection into the left ventricles of the NRMI nude mice (*n* = 7 per group). An in vivo imaging device (IVIS imaging system, PerkinElmer, Massachusetts, UK) was used. Mice were sacrificed after 30 days of tumor cell inoculation. To identify metastatic PCa cells in the bone marrow of recipients after tumor implantation, bone marrow cells from the long bones of the mice were isolated and were stained with HLA-ABC conjugated with fluorescein isothiocyanate (FITC), APC-conjugated Annexin V and 7AAD and were subjected to FACS analysis. (ii) In the second set, 1 × 10^6^ PC-3 cells were subcutaneously implanted into the NRMI nude mice (*n* = 4 mice/group). Tumor samples were collected post-mortem and used for the isolation of tumor-associated myeloid cells and immunohistochemical and immunoblot analysis.

### 4.9. Isolation of Bone Marrow-Derived Cells from the Xenograft Tumors

To isolate tumor-associated bone marrow-derived cells, xenograft tumors were dissected from the NMRI nude mice and were made into single-cell suspensions. The cell suspensions were processed with the EasySep^TM^ Mouse Hematopoietic Progenitor Cell Isolation kit by following the manufacturer’s instructions (Stem Cell Technologies, Vancouver, BC, Canada). Cells that were stained with lineage antigens such as the CD11b and Ly6G/C (Gr1) hematopoietic-myeloid cell markers were obtained. The staining of the cells with the myeloid cell markers including CD11b, CD14 and Gr1 was confirmed using flow cytometry analysis.

### 4.10. Immunoblot Analysis

Immunoblot analysis was performed as previously described [22]. The densitometric quantification of immunoblots was performed using the ImageJ image analysis software (NIH, Baltimore, MD, USA).

### 4.11. Immunohistochemical Analysis

Immunohistochemistry on tumor tissue microarrays was performed as previously described [28]. The staining procedure was performed using a semiautomatic staining machine (Ventana Inc., Oro Valley, AZ, USA). The tumors were fixed in 4% paraformaldehyde for 24 h before decalcification in formic acid and embedded in paraffin sections. Slides were scanned and viewed. Microphotographs were taken by using a high-resolution scanner (ScanscopeCS, Aperio, CA, USA).

### 4.12. Immunofluorescence Analysis

Cell suspensions were fixed on slides in methanol or in 4% paraformaldehyde in PBS. The slides were washed in PBS twice and permeabilized in 0.5% Triton X-100 (Sigma-Aldrich, Stockholm, Sweden) for 10 min at room temperature (RT). The slides were stained with primary antibodies. Primary antibodies including anti-PIP5K1α (Protein Technologies, Manchester, UK) and β-tubulin (Abcam, Cambridge, UK) were used. Secondary antibodies including anti-rabbit conjugated to Alexa Fluor 488 (Invitrogen, Stockholm, Sweden), anti-mouse conjugated to Alexa Fluor 546 (Invitrogen, Stockholm, Sweden) and anti-rabbit conjugated to Rhodamine (Chemicon International Inc, Temecula, CA, USA) were used. Cells were counterstained with DAPI (4′,6-diamidino-2-phenylindole, dihydrochloride). The cells were examined under an Olympus AX70 microscope using the NIS Elements F 2.20 software or a Zeiss Apotome microscope and the Zen 2.3 Lite software (Zeiss, Oberkochen, Germany).

### 4.13. Field-Emission Scanning Electron Microscopic Imaging of PC-3 and U-937 Cells

Mono- or co-cultured cells were cultured on glass coverslips and fixed in 2% paraformaldehyde. The samples were subsequently dehydrated in graded series of ethanol, dried and coated with thin (2 nm) metal film. The morphology of the cells was analyzed by field-emission scanning electron microscopy (FESEM; Zeiss, Oberkochen, Germany) using an in-chamber secondary electron detector at an accelerating voltage of 4 kV and probe current of 120 pA.

### 4.14. Statistical Analysis

Student’s *t*-test was used for statistical analyses of the experimental data. Differences between survival curves were calculated using the log-rank test using a statistical program (SPSS, 16.0). *p*-values lower than 0.05 were considered to be statistically significant.

## 5. Conclusions

Taken together, our study suggests that bone marrow cells play supportive roles, helping PCa cells to establish growth and bone/bone marrow metastasis. Furthermore, PCa cells may utilize tumor-specific signals to adapt to their host microenvironment to gain proliferative and invasive advantages. Therapeutic interventions using PIP5K1α inhibitors may not only inhibit tumor invasion and metastasis but also enhance the host immune system.

## Supplementary Materials

The following are available online at https://www.mdpi.com/2072-6694/12/9/2719/s1, Figure S1: Original Western Blots.
