# Supplementary material for: Establishment of Prostate Tumor Growth and Metastasis Is Supported by Bone Marrow Cells and Is Mediated by PIP5K1α Lipid Kinase
Source: Cancers (Basel). 2020 Sep 22;12(9):2719. doi: 10.3390/cancers12092719 (PMC7564679; doi:10.3390/cancers12092719)

Supplemental data 1 (S1)

Raw data figure 1c

WB Membrane 58

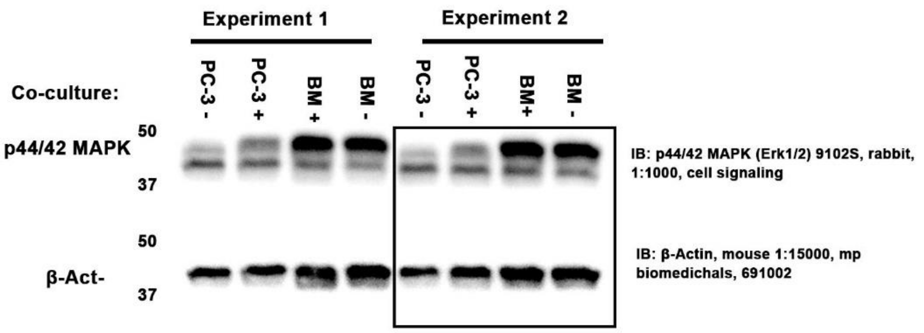

WB Membrane 59

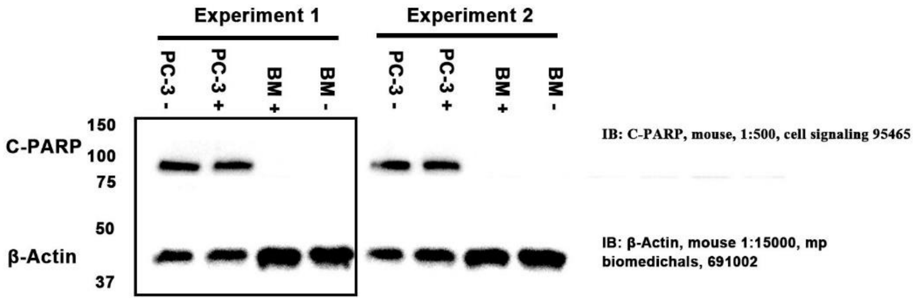

Raw data figure 1e

WB Membrane 22

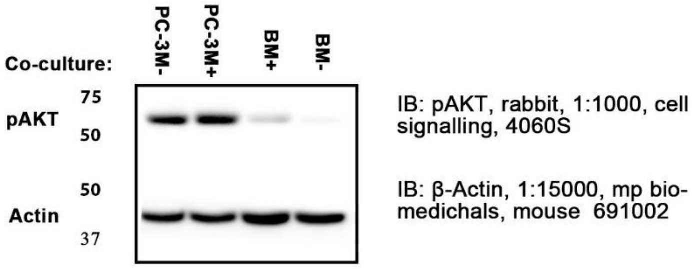

Figure 3c

WB Membrane 65

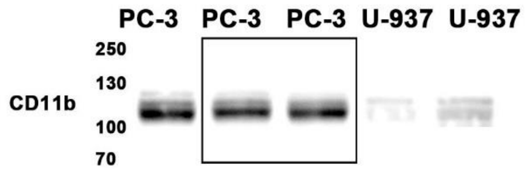

CD11b, 1:500,
Novus Biologicals NB110-89474SS

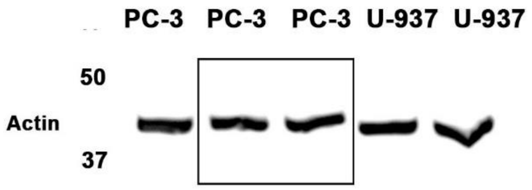

Actin, 1:10000 mouse MP biomedicals 6910002

Raw data Figure 4d

WB membrane 20

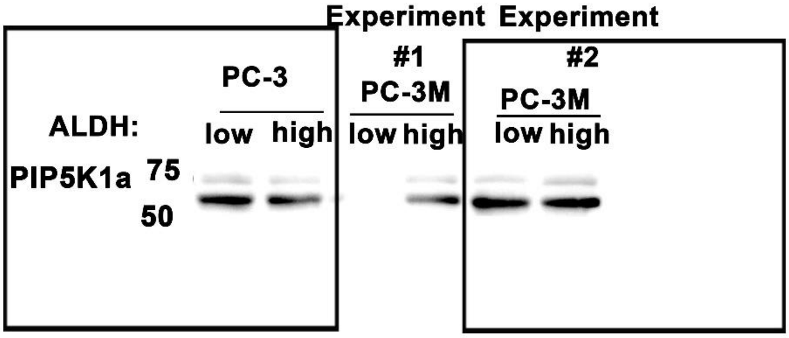

PIP5k1a, rabbit, 1:500, proteintech.

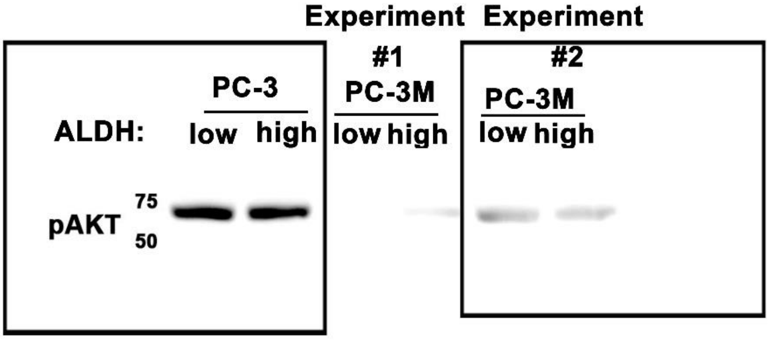

pAKT, rabbit, 1:1000, cell signalling

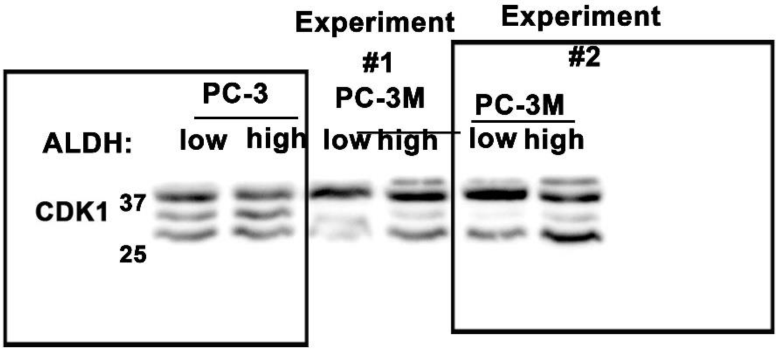

CDK1, rabbit, 1:500, Upstate biotech

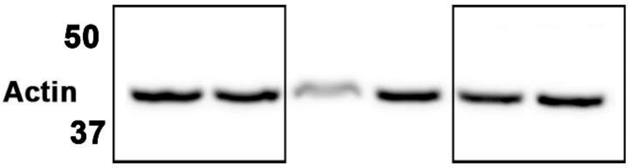

Actin, 1:15000, mp biomedicals

Raw data figure 4e

WB Membrane 5

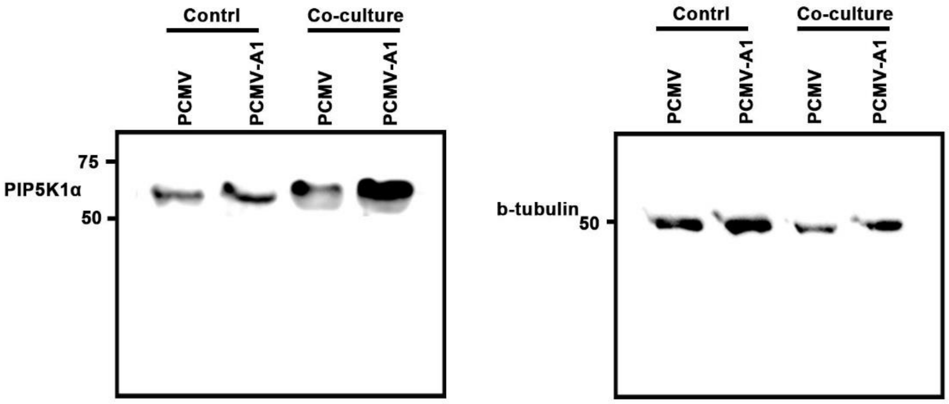

IB: PIP5K1a, rabbit, 1:500, Proteintech, 15713-A-AP

IB: b-tubulin, 1:500, sigma-aldrich, mouse, T4026

Raw data figure 4f

WB Membrane 12

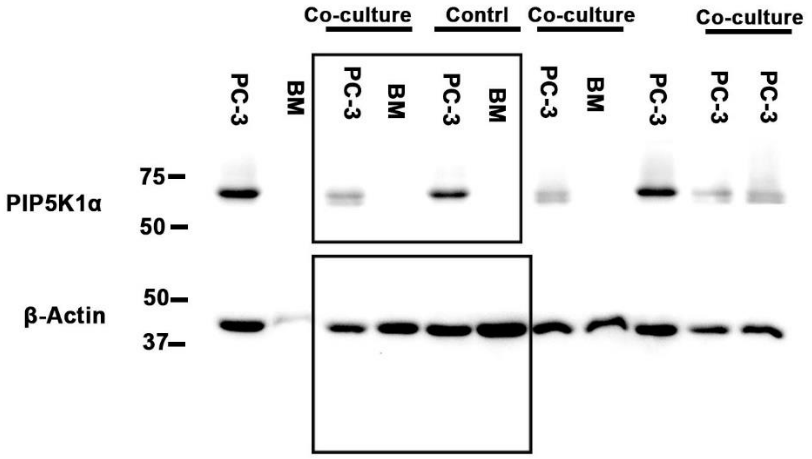

IB: PIP5K1a, rabbit, 1:500, Proteintech, 15713-A-AP

IB: β-Actin, mouse 1:15000, mp biomedichals, 691002

Raw data figure 4g

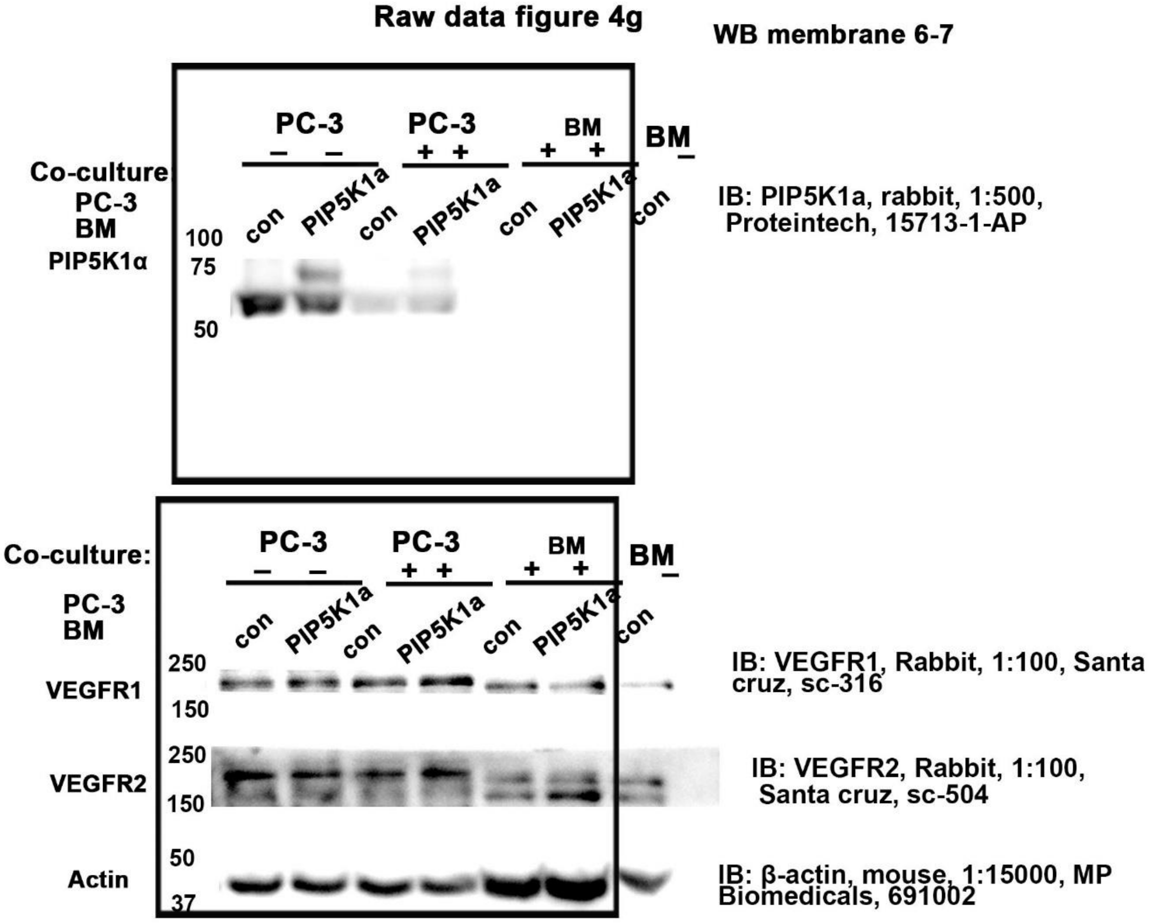

Raw data figure 5b

WB Membrane 1-2

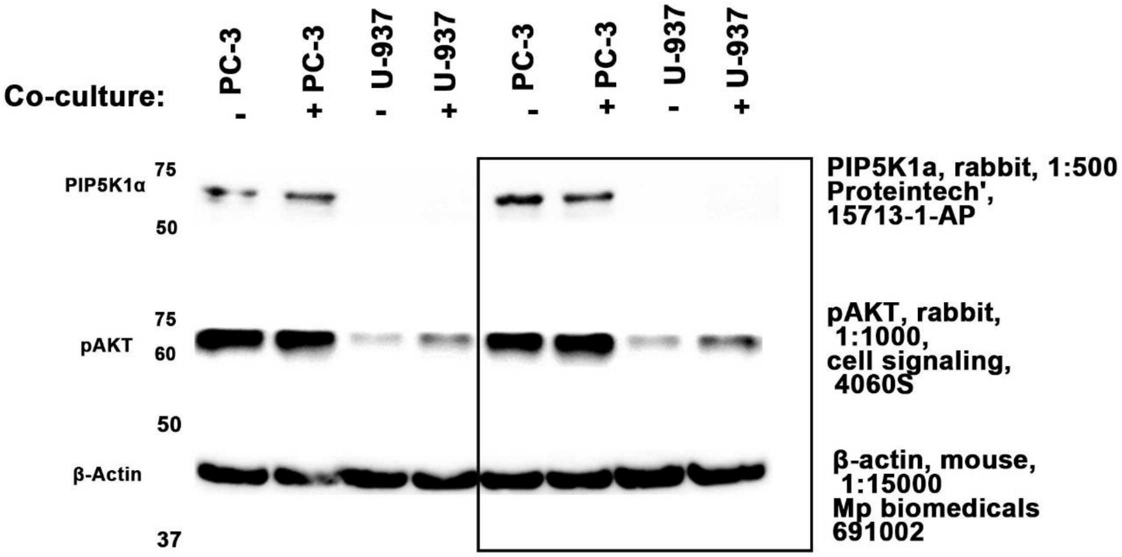

Supplement: Supplementary file 1 [file cancers-12-02719-s001.pdf]
